# Genotype diversity and distribution of *Mycobacterium bovis* from livestock in a small, high-risk area in northeastern Sicily, Italy

**DOI:** 10.1371/journal.pntd.0007546

**Published:** 2019-07-15

**Authors:** Cinzia Marianelli, Benedetta Amato, Maria Beatrice Boniotti, Maria Vitale, Flavia Pruiti Ciarello, Maria Lodovica Pacciarini, Vincenzo Di Marco Lo Presti

**Affiliations:** 1 Department of Food Safety, Nutrition and Veterinary Public Health, Istituto Superiore di Sanità, Rome, Italy; 2 Sezione Diagnostica Barcellona P.G., Istituto Zooprofilattico Sperimentale della Sicilia, Barcellona Pozzo di Gotto, Italy; 3 National Reference Centre for Bovine Tuberculosis, Istituto Zooprofilattico Sperimentale della Lombardia e dell’Emilia Romagna, Brescia, Italy; Hospital Infantil de Mexico Federico Gomez, UNITED STATES

## Abstract

Bovine tuberculosis (bTB) caused by *Mycobacterium bovis* is an important re-emerging disease affecting livestock, wildlife and humans. Epidemiological studies are crucial to identifying the source of bTB infection, and its transmission dynamics and host preference, and thus to the implementation of effective strategies to contain it. In this study, we typed *M*. *bovis* isolates from livestock, and investigated their genetic diversity and distribution. A total of 204 *M*. *bovis* isolates were collected from cattle (n = 164) and Sicilian black pigs (n = 40) reared in a limited area of the province of Messina, northeastern Sicily, an area that had previously been identified as having the highest incidence of bTB in livestock on the island. All *M*. *bovis* isolates were typed by both spoligotyping and 12-loci MIRU-VNTR analysis. Results from both methods were then combined in order to improve the discriminatory power of *M*. *bovis* typing. We identified 73 combined genetic profiles. Thirty-five point six percent of the profiles were common to at least two animals, whereas 64.4% of profiles occurred in only one animal. A number of genetic profiles were predominant in either cattle or black pigs. We identified common genetic patterns in *M*. *bovis* isolates originating not only from neighboring districts, but also from non-neighboring districts. Our findings suggest that bTB is widespread in our setting, and is caused by a large number of genetically diverse *M*. *bovis* strains. The ecology and farming practices characteristic of the area may explain the substantial *M*. *bovis* heterogeneity observed, and could represent obstacles to bTB eradication.

## Author summary

Bovine tuberculosis is a widespread infectious disease affecting both domestic and wild animals, as well as humans. In addition to being of public health concern, the disease, caused mainly by *Mycobacterium bovis*, has a significant economic impact on the farming industry due to the costs of eradication efforts. In Sicily, the largest of the Italian islands, bovine tuberculosis in livestock is of great concern, and targeted control strategies are needed. Molecular epidemiology is an essential tool for determining the distribution of a disease, so as to control it and minimize its threat to the population. We typed *M*. *bovis* isolates isolated from cattle and pigs reared in a limited area of Sicily. An in-depth comparison of the genetic makeup of these isolates allowed us a better understanding of the genetic diversity and distribution of the pathogen in our population of animals. We found that the disease is widespread in the area and caused by a large variety of *M*. *bovis* strains, which are in several cases common to different species of livestock. The paper concludes with a discussion of the findings in light of the environmental and ecological setting, and of farming practices in the area. The results are expected to contribute to the improvement of surveillance and control programs of bovine tuberculosis in the region.

## Introduction

Bovine tuberculosis (bTB) is an infectious disease of worldwide distribution caused mainly by *Mycobacterium bovis*, one of the members of the *Mycobacterium tuberculosis* complex (MTC).

*M*. *bovis* has the broadest host range of any member of the MTC, and an intricate epidemiological pattern of infection. The pathogen may infect a wide range of domestic animal species, with negative impacts on both animal productivity and the international trade of animal products. *M*. *bovis* can spread via aerosols, suckling, and the sharing of water and feed [1,2]. *M*. *bovis* may also spread to wildlife species which can act as reservoir hosts, contributing to the transmission and persistence of the disease [3,4]. Finally, it may infect humans, causing a disease–zoonotic tuberculosis—which is indistinguishable from that caused by *M*. *tuberculosis* [5,6]. Direct contact with infected animals and the consumption of unpasteurized dairy products have been indicated as the most likely routes of zoonotic transmission [7].

In most industrialized countries, animal test-and-slaughter schemes have successfully reduced the occurrence of bTB, and only occasional cases of *M*. *bovis* infection in humans are documented [8]. *M*. *bovis* represents a serious public health issue in non-industrialized countries, however, where factors such as absent or inadequate bTB control programs, immunodeficiency, close contact with infected animals, consumption of infected animal products and malnutrition, contribute to increase the risk of zoonotic tuberculosis [8,9].

The design of intervention strategies in animals is informed mainly by the epidemiology of the disease. Comprehensive epidemiological studies of bTB can provide valuable insights into the sources of infection, routes of transmission, geographical localization, host preference, disease dynamics and risk factors for the maintenance and spread of the disease, thus contributing to contain the disease in animals and reduce the risk to humans [2].

Various molecular-based techniques have been developed to study the epidemiology of *M*. *bovis* infections [2]. Spoligotyping [10] and mycobacterial interspersed repetitive unit-variable number tandem repeats (MIRU-VNTR) typing [11,12] are the most commonly used methods for *M*. *bovis* genotyping. The use of spoligotyping in combination with MIRU-VNTR typing has been shown to improve the discriminatory power of *M*. *bovis* typing [13–15]. Combined genetic profiles have recently been used to analyze the transmission of *M*. *bovis* in France [16], Midwest Brazil [17], Cameroon [18] and Mozambique [19].

In Italy, a national eradication program has resulted in a gradual reduction of bTB prevalence in most regions. On the island of Sicily, bTB remains a major concern, however. Our group has previously investigated the role played by the Sicilian black pig—an autochthonous free- or semi-free-ranging breed of domestic pig—in the maintenance of bTB in two neighboring areas in northeastern Sicily: the Nebrodi and Madonie Natural Parks [20]. The characteristics of the lesions, their localization and the genetic profiles of *M*. *bovis* isolates indicated that Sicilian black pigs may act as a reservoir of bTB in the ecological setting studied [20]. More recently, our group has genotyped MTC isolates from livestock and wild animals throughout Sicily [21]. We found evidence of considerable diversity of *M*. *bovis* spoligotypes and MIRU-VNTR profiles in domestic animals, with the province of Messina recording the highest number of outbreaks in the period 2004–2014 in livestock in Sicily [21]. The latest Epidemiological Veterinary Bulletin of the province of Messina, published in December of 2018 (https://docs.google.com/viewer?a=v&pid=sites&srcid=aXpzc2ljaWxpYS5pdHxpenN8Z3g6NzIzNjhmOTYwYWJjMDEzMw), reported bTB prevalence (1.75%) and incidence (1.47%) data for the province, in terms of the percentage of farms affected. During recent bTB control campaigns in the province of Messina, Caronia has emerged as one of the districts with the highest incidence rates of bTB in livestock. In-depth epidemiological studies aimed at investigating genetic relationships among *M*. *bovis* isolates in animals in this high-risk area, especially in the district of Caronia, may contribute to more effective bTB eradication interventions.

In this study, we typed *M*. *bovis* isolates from livestock—cattle and Sicilian black pigs—reared in the province of Messina in Sicily, by both spoligotyping and MIRU-VNTR typing, for epidemiological purposes.

## Methods

### Ethics statement

Tissue samples from slaughtered cattle and Sicilian black pigs revealing tubercular lesions on postmortem examination were collected at abattoirs. Inspections at abattoirs were carried out in accordance with Italian law, and no permission from abattoir owners was needed.

### Source and geographic origin of *M*. *bovis* isolates

Over a period of two years, from 2015 to 2016, tissue samples from slaughtered cattle and Sicilian black pigs revealing tuberculous-like lesions on *postmortem* examination were collected at the only three abattoirs allowed to slaughter bTB-infected animals in the province of Messina: Pascoli dei Nebrodi soc. coop 1503 M Mirto, Caruso Impex 1344 M Barcellona Pozzo di Gotto, and Si.L.Car. 940 M CEE Merì. Slaughtered animals included both those for consumption purposes and those with positive response to bovine tuberculin within the frame of bTB control and eradication programs. Tissue samples were cultured and isolates were typed as previously described [20]. Of all isolates identified as *M*. *bovis* over the two years (N = 765) studied, a total of 204 *M*. *bovis* isolates were randomly selected for analysis from slaughtered cattle (n = 164) and Sicilian black pigs (n = 40).

The province of Messina (3,247 km^2^) covers approximately 11% of the total area of the island. This largely mountainous province, divided into 108 districts, is home to the Nebrodi Park, a rural nature reserve extending over an area of nearly 860 km^2^. This mostly mountainous park is covered by wide pastures and woods that give shelter to numerous species of wild mammals, birds, reptiles, and invertebrates. Wild boar are absent from the Nebrodi Park. The Sicilian black pig lives mostly in the woods of the Nebrodi Park, where it is reared in free or semi-free roaming conditions, frequently sharing pastures with cattle. Approximately 1,800 cattle and 1,200 black pig herds are present in the province of Messina.

Sample data concerning the name of the farmer, farm location (district of the Messina province) and main type of livestock reared on the farm, were recorded for every slaughtered animal with a culture-confirmed diagnosis of bTB.

### Spoligotyping and MIRU-VNTR analysis of *M*. *bovis* isolates

All *M*. *bovis* isolates were typed by both spoligotyping [10] and 12-loci MIRU-VNTR analysis. In spoligotyping, the spacer sequences contained in the direct repeat locus were detected by hybridization onto a spoligotyping membrane (Ocimum Biosolutions, Hyderabad, India). The spoligotypes obtained were checked against an international spoligodatabase (Mbovis.org Mycobacterium bovis Spoligotype Database https://www.mbovis.org/).

For MIRU-VNTR typing, 12 genomic loci were selected, according to Boniotti and colleagues [13], and amplified individually: VNTR loci 2165, 2461, 0577, 0580 and 3192 (i.e., ETR-A to–E) [22], VNTR locus 2996 (i.e., MIRU26) [23], VNTR loci 2163a, 2163b, 3155 and 4052 [24], and VNTR loci 1895 and 3232 [25]. *M*. *tuberculosis* H37Rv was used as reference strain. Allele assignment was performed on the basis of PCR fragment size as compared to a 50-bp molecular weight marker.

The resulting genetic profiles, obtained by combining spoligotypes and MIRU-VNTR results, were used for the epidemiological investigation.

### Clustering analysis

Minimum spanning tree analysis based on 12-locus MIRU-VNTR results was used to infer relationships between the isolates sharing the same spoligotype. The number of isolates assigned to each combined genetic profile (spoligotype and MIRU-VNTR type), the host (cow or Sicilian black pig) and the Messina district of pathogen isolation, are provided in the graphs. Data analysis was performed using BioNumerics Seven platform (Applied Maths, Sint-Martens-Latem, Belgium). Applied Maths has granted us a temporary BioNumerics evaluation license and authorized the publication of the results.

## Results

### Cases of bTB among animals

A total of 204 *M*. *bovis* isolates from slaughtered cattle (n = 164) and Sicilian black pigs (n = 40) with bTB were anaysed.

**Fig 1:**
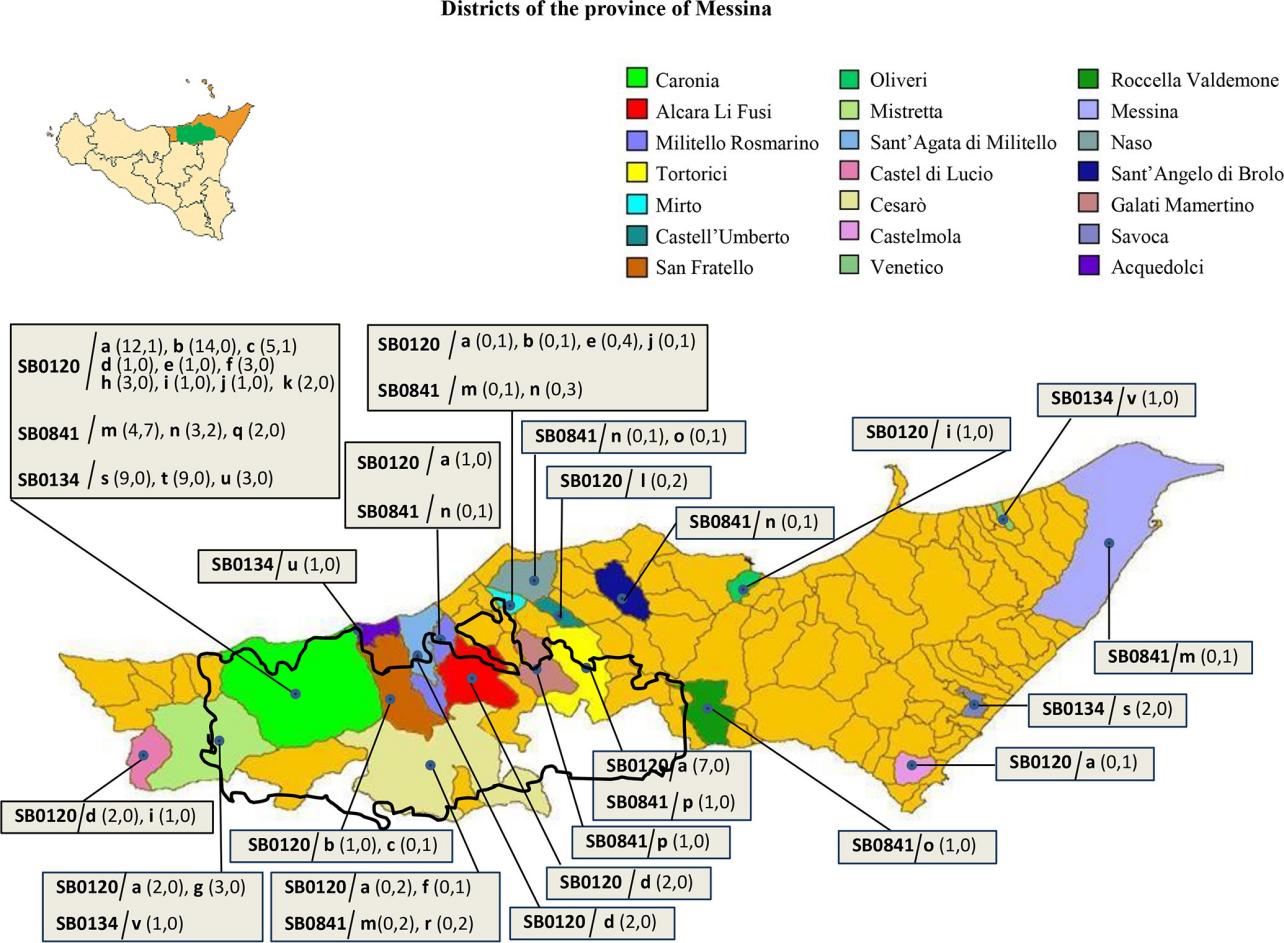
The geographical area under study: Combined genetic profiles of *M*. *bovis* isolates by district. The island of Sicily, with the province of Messina (in orange) and the Nebrodi Natural Park (in green) is shown in the upper left corner. The large map below shows the province of Messina in detail, the boundaries of the Nebrodi Park and the most frequent combined genetic profiles of *M*. *bovis* isolates by district. The number of isolates for each profile is indicated in brackets: the first number corresponds to the isolates from cows and the second to those from black pigs. The map was adapted from: https://commons.wikimedia.org/wiki/File:Map_of_comune_of_Messina_(metropolitan_city_of_Messina,_region_Sicily,_Italy).svg, public domain.

Bovine tuberculosis cases originated from a total of 114 small-scale herds in 21 out of the province's 108 districts (Fig 1). Of the infected farms, 96 were cattle farms, 16 were black pig farms and two farms bred both cattle and black pigs. In 76 of 114 small-scale farms (66.5%), a single isolate was found; in 18 farms (16%), two isolates, were identified, with two different genotypes in most of these herds (14/18); and in 20 farms (17.5%), more than two isolates were found, with at least two different genotypes in most herds (12/20).

### Genotyping of *M*. *bovis* isolates

All *M*. *bovis* isolates were genotyped by both spoligotyping and 12-loci MIRU-VNTR analysis. Results are shown in Table 1.

**Table 1:** Spoligotypes and MIRU-VNTR types of *M*. *bovis* isolates (N = 204).

Host (n)	Spoligotype (n_c_, n_p_)	MIRU-VNTR type												
		Code *2165 2461 0577 580 3192 2163a 2163b 4052 1895 3155 3232 2996* (n_c_, n_p_)												
cattle (164) black pigs (40)	SB0120 (85, 17) SB0841 (21, 23) SB0134 (32, 0) SB0850 (5, 0) SB2473 (5, 0) SB0121 (3, 0) SB0822 (3, 0) SB0133 (2, 0) SB1564 (2, 0) SB0961 (1, 0) SB1305 (1, 0) SB1565 (1, 0) SB1566 (1, 0) SB1570 (1, 0) SB1572 (1, 0)													
		4	5	5	3	3	10	4	4	4	3	6	5	(22, 5)
		-	3	-	-	-	-	-	-	-	-	-	-	(1, 0)
		-	4	-	-	-	-	-	-	-	-	-	-	(2, 0)
		-	-	-	-	-	7	-	-	-	-	-	-	(3, 0)
		-	-	-	-	-	-	3	-	-	-	-	-	(1, 0)
		-	-	-	-	-	-	5	-	-	-	-	-	(1, 0)
		-	-	-	-	-	-	-	2	-	-	-	-	(3, 0)
		-	-	-	-	-	-	-	3	-	-	-	-	(5, 1)
		-	-	-	-	-	-	-	-	2	-	-	-	(2, 4)
		-	-	-	-	-	-	-	-	-	-	-	4	(1, 0)
		-	-	-	-	-	-	3	-	2	-	-	-	(1, 0)
		-	-	-	-	-	-	-	-	3	-	5	-	(5, 0)
		2	3	-	-	-	-	-	-	-	-	-	-	(1, 0)
		3	3	-	-	-	-	-	-	-	-	-	-	(7, 2)
		3	3	-	-	-	-	3	-	-	-	-	-	(1, 0)
		3	3	-	-	-	-	6	-	-	-	-	-	(15, 1)
		3	3	-	-	-	-	-	-	-	-	7	-	(1, 0)
		3	3	-	-	-	-	6	2	-	-	-	-	(1, 0)
		3	3	-	-	-	-	6	-	-	2	-	-	(1, 0)
		3	3	-	-	-	-	6	-	-	-	7	-	(1, 0)
		3	3	-	-	-	-	-	-	-	-	-	4	(1, 1)
		5	-	-	-	-	-	-	-	-	-	-	-	(6, 8)
		5	-	-	-	-	5	-	-	-	-	-	-	(1, 1)
		5	-	-	-	-	-	-	1	-	-	-	-	(1, 0)
		5	-	-	-	-	-	-	-	-	-	5	-	(4, 12)
		5	-	-	-	-	-	-	-	-	-	-	6	(1, 0)
		5	-	-	-	-	-	-	-	-	1	5	-	(0, 1)
		5	-	-	-	-	-	-	-	-	-	5	4	(0, 2)
		5	-	-	-	-	-	-	-	-	-	5	6	(2, 0)
		5	-	-	-	4	-	-	1	-	-	-	-	(1, 0)
		5	-	-	-	4	-	-	5	-	-	-	-	(7, 0)
		5	-	-	-	4	-	-	5	-	2	-	-	(1, 0)
		5	-	-	-	-	-	3	-	-	-	5	-	(1, 0)
		5	-	2	-	-	-	-	-	-	-	-	4	(1, 0)
		5	-	6	-	-	-	-	-	-	-	5	-	(1, 0)
		5	-	6	-	-	-	-	-	-	-	8	-	(1, 0)
		5	3	-	-	-	-	-	-	-	-	-	-	(1, 0)
		5	3	-	-	-	-	-	-	3	-	-	-	(0, 2)
		5	3	-	-	4	-	-	1	-	-	-	-	(1, 0)
		5	3	-	-	4	-	-	5	-	-	-	-	(3, 0)
		5	4	-	-	-	-	-	-	-	-	-	-	(3, 0)
		5	4	-	-	-	7	-	-	-	-	-	-	(1, 0)
		5	4	-	-	-	-	-	1	-	-	-	-	(1, 0)
		5	4	-	-	-	-	-	3	-	-	-	-	(1, 0)
		5	4	-	-	-	-	-	-	-	1	-	-	(3, 0)
		5	4	-	-	-	-	-	-	-	-	7	-	(1, 0)
		5	4	-	-	-	-	-	-	-	1	7	-	(2, 0)
		5	4	-	-	-	-	3	5	-	-	-	-	(1, 0)
		5	4	-	-	4	-	-	5	-	-	-	-	(9, 0)
		5	4	-	-	4	-	-	5	-	-	-	7	(1, 0)
		5	4	-	2	4	-	-	5	-	-	-	-	(2, 0)
		5	4	-	-	4	-	2	5	-	-	-	-	(4, 0)
		5	4	-	-	4	-	3	5	-	-	-	-	(11, 0)
		5	4	-	-	4	-	-	5	-	-	8	-	(1, 0)
		5	4	-	2	4	-	-	5	-	-	5	-	(1, 0)
		5	4	-	-	4	-	3	6	-	2	-	-	(1, 0)
		5	4	-	2	4	-	-	5	-	-	5	6	(1, 0)
		6	4	-	-	-	-	-	-	-	-	-	6	(1, 0)
		6	4	-	-	-	-	2	5	-	-	-	2	(1, 0)
		6	4	3	-	-	-	2	5	-	-	7	2	(6, 0)
		6	4	3	-	-	-	2	5	-	-	9	2	(2, 0)
		6	4	3	-	-	-	2	9	-	-	9	2	(1, 0)

n = number of isolates; n_c_ = number of isolates from cattle; n_p_ = number of isolates from black pigs. MIRU-VNTR codes derive from 12 genomic loci (see Materials and Methods). Dashes indicate that the code does not differ from the one written in full.

A total of 15 spoligotypes were found. All 15 genotypes were present in *M*. *bovis* cattle isolates, with SB0120, SB0134 and SB0841 being the most prevalent profiles (85, 32 and 21 isolates, respectively). *M*. *bovis* pig isolates, on the other hand, belonged to only two of the 15 genotypes–SB0841 and SB0120 and (23 and 17 isolates, respectively). Overall, SB0120, SB0841 and SB0134 were the most frequent spoligotypes circulating in livestock, accounting for 50% (102/204), 21.6% (44/204) and 15.7% (32/204) of *M*. *bovis* isolates, respectively.

MIRU-VNTR analysis yielded a total of 62 MIRU-VNTR types: 50 types in cattle only, three types in pigs only, and nine types common to both animal sources. The profiles MIRU-VNTR-a (22 isolates), MIRU-VNTR-b (15 isolates) and MIRU-VNTR-s (11 isolates) were the most frequently identified in cattle. MIRU-VNTR-m (12 isolates) and MIRU-VNTR-n (8 isolates) were predominant in pigs. Overall, MIRU-VNTR-a, MIRU-VNTR-b, MIRU-VNTR-m and MIRU-VNTR-n were the most frequent MIRU-VNTR types found in livestock, accounting for 13.2% (27/204), 7.8% (16/204), 7.8% (16/204) and 6.9% (14/204) of *M*. *bovis* isolates, respectively.

The combination of both typing methods increased the number of genetic profiles to 73 (S1 Table). Specifically, 60 profiles affected cattle only, four profiles affected black pigs only and nine profiles affected both populations. In addition, while most profiles (47/73, 64.4%) affected a single animal, the remaining profiles (26/73, 35.6%) were common to at least two bTB-infected animals.

### Epidemiological investigation

**Fig 2:**
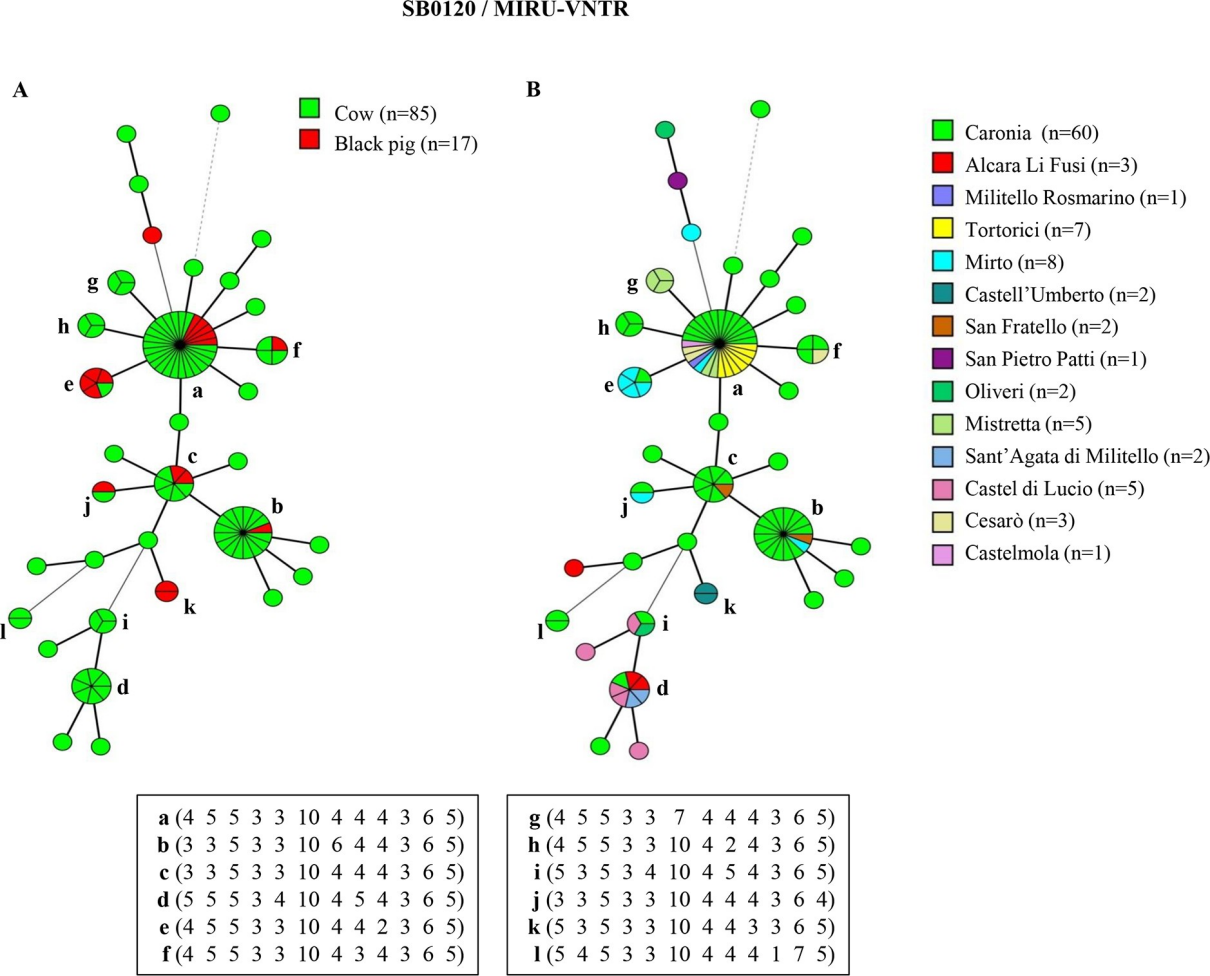
Minimum spanning trees of 102 *M*. *bovis* isolates with spoligotype SB0120 based on 12-locus MIRU-VNTR analysis. The total number of isolates is indicated in the legend, in parentheses. Each circle represents a distinct 12-locus MIRU-VNTR type. The size of circles is proportional to the number of isolates, indicated as segments within each circle. MIRU-VNTR profiles with two strains or more are marked (a-l). The colors within the circles represent the hosts–cow or black pig—in panel A, and the district in panel B.

**Fig 3:**
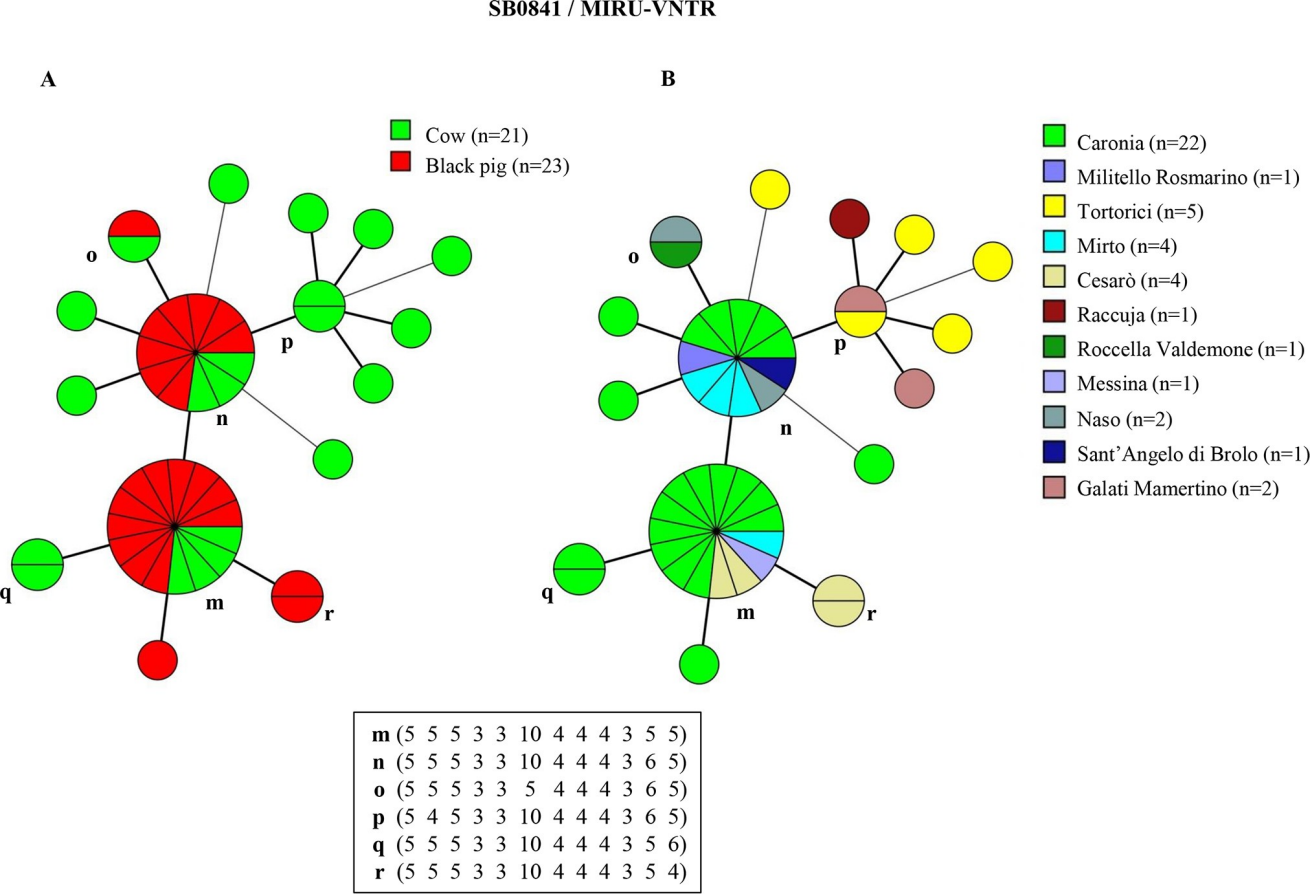
Minimum spanning trees of 44 *M*. *bovis* isolates with spoligotype SB0841 based on 12-locus MIRU-VNTR analysis. The total number of isolates is indicated in the legend, in parentheses. Each circle represents a distinct 12-locus MIRU-VNTR type. The size of circles is proportional to the number of isolates, indicated as segments within each circle. MIRU-VNTR profiles with two strains or more are marked (a-l). The colors within the circles represent the hosts–cow or black pig—in panel A, and the district in panel B.

**Fig 4:**
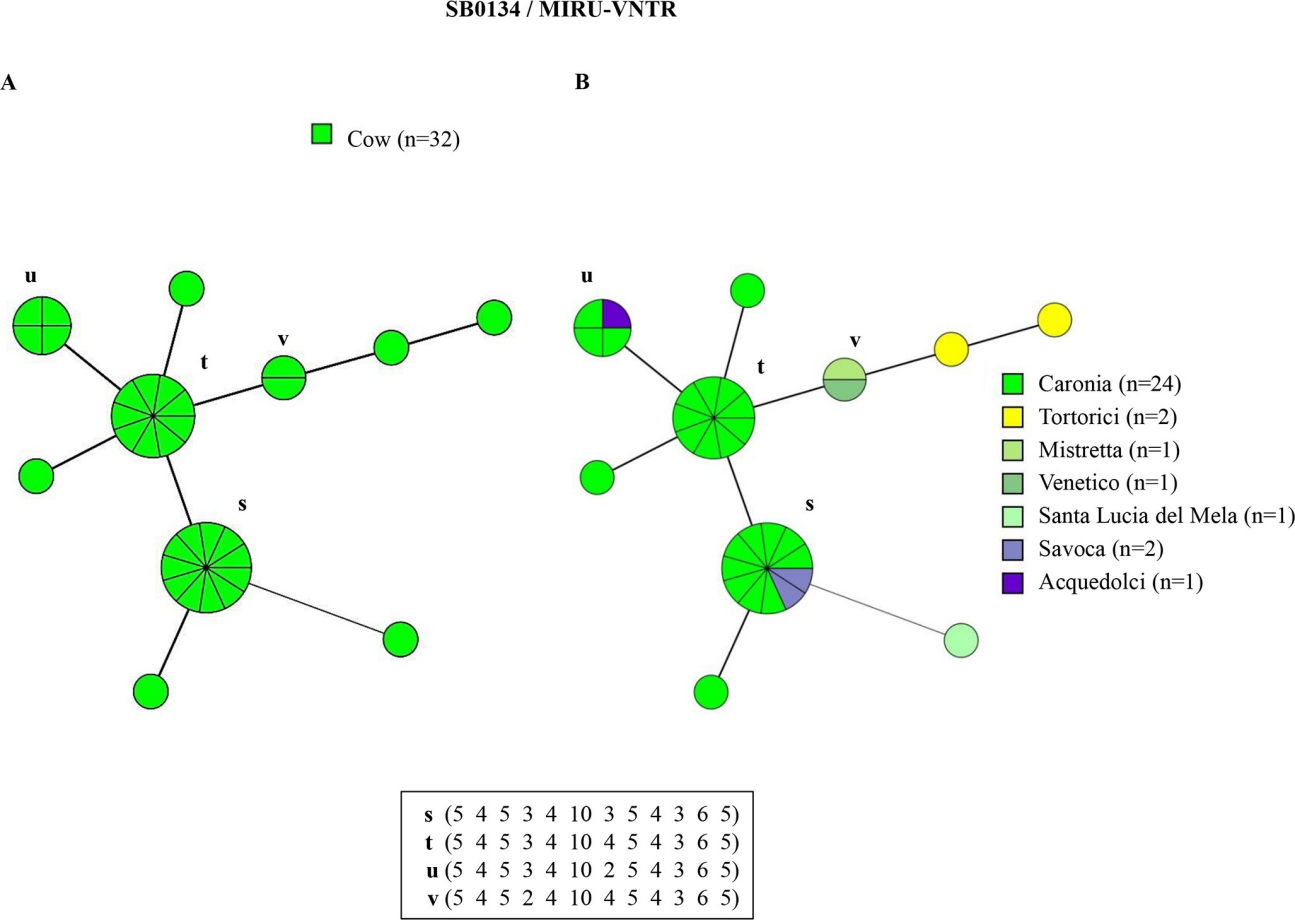
Minimum spanning tree of *M*. *bovis* isolates in cattle with spoligotype SB0134 based on 12-locus MIRU-VNTR analysis. The total number of isolates is indicated in the legend, in parentheses. Each circle represents a distinct 12-locus MIRU-VNTR type. The size of circles is proportional to the number of isolates, indicated as segments within each circle. MIRU-VNTR profiles with two strains or more are marked (a-l). The green color within the circles represent infected cows in panel A. The colors within the circles in panel B represent the district.

The combined genetic profiles obtained by subtyping the predominant spoligotypes SB0120, SB0841 and SB0134 with 12-loci MIRU-VNTR analysis, were used for the epidemiological investigation. Results, together with information concerning the animal host and the district of isolation, are shown in Figs 2–4. For a complete list of identified profiles, see supporting information (S1 Fig, S2 Fig and S3 Fig).

Thirty-three genetic profiles were found by subtyping the SB0120 spoligotype with MIRU-VNTR (Fig 2). Common profiles identified in at least two *M*. *bovis* isolates were marked (SB0120/a-l). The most frequent combined genetic profiles were SB0120/a (27 isolates: 22 from cows and 5 from pigs), SB0120/b (16 isolates: 15 from cows and 1 from a pig), SB0120/c (7 isolates: 5 from cows and 2 from pigs) and SB0120/d (7 isolates: all from bovine hosts), accounting for 13.2% (27/204), 7.8% (16/204), 3.4% (7/204) and 3.4% (7/204) of *M*. *bovis* isolates, respectively (Fig 2, panel A). SB0120/a occurred in seven districts, SB0120/b in three districts, SB0120/c in two districts, and SB0120/d in four districts (Fig 2, panel B).

Sixteen genetic profiles were obtained by combining the SB0841 spoligotype and MIRU-VNTR results (Fig 3). Profiles found in two animals or more were marked (SB0841/m-r). The most frequent combined profiles were SB0841/m (15 isolates: 4 from cows and 11 from pigs) and SB0841/n (11 isolates: 3 from cows and 8 from pigs), accounting for 7.3% (15/204) and 5.4% (11/204) of *M*. *bovis* isolates, respectively (Fig 3, panel A). Profiles SB0841/m and SB0841/n occurred in four districts, and five districts, respectively (Fig 3, panel B).

Ten genetic profiles were obtained by combining the SB0134 spoligotype and MIRU-VNTR results (Fig 4). Profiles found in two animals or more were similarly marked (SB0134/s-v). Unlike SB0120/MIRU-VNTR and SB0841/MIRU-VNTR types, SB0134/s-v profiles were found only in cattle. The most frequent combined genetic profiles were SB0134/s (11 isolates) and SB0134/t (9 isolates), accounting for 5.4% (11/204) and 4.4% (9/204) of isolates, respectively (Fig 4, panel A). Profiles SB0134/s and SB0134/t occurred in two districts and one district, respectively (Fig 4, panel B).

The distribution and frequency of the SB0120/a-l, SB0841/m-r and SB0131/s-v combined profiles across the province of Messina are shown in Fig 1.

The fact that the highest number of bTB cases, in both cattle and pigs, was recorded in Caronia (116/204; 56.7% of *M*. *bovis* isolates), was probably due to the fact that the highest number of slaughtered animals were sampled from this district.

Identical genetic profiles were detected in livestock originating from neighboring districts (Fig 1) such as those of Caronia, Mistretta and Cesarò (SB0120/a), Caronia and Cesarò (SB0120/f; SB0841/m), Caronia and San Fratello (SB0120/b; SB0120/c), Mirto and Naso (SB0841/n—in pigs only), Galati Mamertino and Tortorici (SB0841/p—in cows only), and Caronia and Acquedolci (SB0134/u—in cows only).

Interestingly, non-neighboring districts—some of which are quite distant from one another—exhibited identical profiles as well (Fig 1). For example, SB0120/a was detected in the districts of Caronia, Castelmola, Tortorici, Mirto, Militello Rosmarino and Cesarò, SB0841/m in the districts of Caronia, Mirto and Messina, and SB0134/v in the districts of Venetico and Mistretta.

Different source animals were also found to carry identical profiles. Profiles SB0120/a, SB0120/b, SB0120/e, SB0120/j, SB0841/m, AB0841/n and SB0841/o were detected in both cattle and black pigs; profiles SB0120/d, SB0120/i, SB0134/s and SB0134/v were instead recorded in cattle only.

As mentioned above, profiles affecting a single animal were also detected: SB0120/g, SB0120/h, SB0120/k, SB0841/q and SB0841/t in cattle, SB0120/l and SB0841/r in pigs.

## Discussion

The present study aimed at investigating the genetic diversity and distribution of *M*. *bovis* in livestock (cattle and black pigs) in an area, the province of Messina, identified as having the highest bTB incidence in Sicily. We collected and typed 204 *M*. *bovis* isolates from slaughtered animals with tuberculous-like lesions. We then combined spoligotyping and 12-loci MIRU-VNTR analysis results in order to increase the discriminatory power of *M*. *bovis* typing.

Studies have shown that combined genotyping, such as spoligotyping with MIRU-VNTR, affords greater discriminatory power of *M*. *bovis* typing than the use of either method alone [13–15]. Combined genetic profiles have recently been used to study the molecular epidemiology of *M*. *bovis* in vast areas in France [16], Midwest Brazil [17], Cameroon [18] and Mozambique [19]. The present study is a detailed examination of the genetic diversity of *M*. *bovis* in a small, high-risk area.

We combined spoligotypes and MIRU-VNTR typing, and used the resulting genetic profiles to perform a detailed assessment of *M*. *bovis* epidemiology in a small geographical area. Our results showed high genetic diversity among *M*. *bovis* isolates from across the province of Messina. Numerous genetic profiles were common to cattle and/or black pigs from both neighboring and distant districts, suggesting intense intra- and inter-species *M*. *bovis* transmission in the area under study.

SB0120—the most common *M*. *bovis* spoligotype worldwide [26], and one that has been shown by our group to be the most prevalent in Sicily as a whole [20,21]—was confirmed here as the predominant spoligotype in the province of Messina as well.

While 15 spoligotypes were identified in *M*. *bovis* cattle isolates, only two—SB0120 and SB0841—were revealed in *M*. *bovis* black pig isolates. Based on the social behavior and eating habits (hunting and rooting) of pigs in general, and on the fact that free- or semi-free-range farming is the norm for Sicilian black pigs, one might have expected to find as large a variety of spoligotypes among pigs as that observed in cattle, or more. Instead, we characterized a much smaller number of spoligotypes in pigs than in cattle. These findings are in agreement with our previous studies, where a large variety of spoligotypes were identified in cattle [21], while in Sicilian black pigs, the spoligotypes found were almost exclusively SB0120 and SB0841 [20,21]. All the above findings seem to indicate that cattle and black pigs in our setting vary in their susceptibility to infection with specific *M*. *bovis* spoligotypes, although a larger sample of black pigs should be studied to confirm or refute this hypothesis.

We identified 73 different combined genetic profiles in the province of Messina. These profiles were distributed across 114 small-scale herds and 21 different districts. Most of the *M*. *bovis* isolates included in our study were from Caronia. This is consistent with the high bTB incidence rate found in the course of recent bTB control campaigns in Sicily, which resulted in large-scale slaughtering. The greater *M*. *bovis* variability identified in Caronia, as compared to other districts, is probably due to the fact that the number of bTB-infected animals sampled from this district was the highest. Of the profiles identified, 35.6% were common to at least two animals, whereas 64.4% occurred in only one animal. Some genetic profiles were predominant in either the cattle or the black pig population (e.g. SB0120/a, SB0120/b and SB0134/s in cattle and SB0841/m and SB0841/n in black pigs). We discovered that animals sharing *M*. *bovis* genetic profiles could originate not only from the same farm, but also from farms located in neighboring and even non-neighbouing districts. Taken together, the above findings suggest that bTB is widespread in our setting and is caused by a large number of genetically diverse *M*. *bovis* strains distributed across the province.

Compared to our previous study showing the distribution of different spoligotypes throughout Sicily over a period of 10 years [21], here we have provided a close-up look at the genotype distribution of combined genetic profiles in the province of Messina, hoping to contribute to the improvement of bTB surveillance and control programs in this high risk area.

Circumstances known to favor bTB transmission include the presence of undiagnosed infected animals, fence-line contact with other herds and animals in close living quarters [27–29]. In addition, the geography and ecology of the province of Messina, as well as local farming practices may contribute to explain the spread of genetically diverse *M*. *bovis* strains across the province. The area is largely mountainous, and numerous small-scale farms are scattered throughout the province. Several of the districts studied are situated in the Nebrodi Park (Acquedolci, Alcara Li Fusi, Caronia, Cesarò, Galati Mamertino, Militello Rosmarino, Mistretta, Sant’Agata Militello, San Fratello and Tortorici).

Cattle and sheep are also bred in the park, and frequently share pastures with black pigs. Moreover, herds of cattle and sheep are moved between summer and winter pastures, a traditional animal husbandry practice known as transhumance. These farming methods may be expected to increase the probability of both inter-species and intra-species contagion, even between distant farms. Bovine tuberculosis-affected animals may shed mycobacteria in the ecosystem, contaminate food, water and pastures, and thus transmit the disease to both livestock and wildlife. Direct and/or indirect contact between infected and uninfected animals (livestock-livestock, livestock-wildlife, wildlife-livestock) may occur at shared pastures, waterholes and feeding sites, especially in the dry season.

Local livestock trade and the exchange of stud animals are also common in Sicily, and may facilitate the spread of infection to other farms and districts.

In conclusion, bTB is a major concern in Sicily, especially in the province of Messina. We found a large number of genetically different *M*. *bovis* strains causing the disease in livestock bred in a small geographical area. Numerous common genetic patterns were identified in farm animals from neighboring and even relatively distant districts. Factors involving the local environment, ecology and farming management practices may explain the high level of *M*. *bovis* heterogeneity we observed in both cattle and black pigs, and are likely to represent obstacles to bTB eradication. This information may contribute to bTB prevention efforts through increased controls at farms, especially during local livestock trade and exchange of stud animals, or increased vigilance on the part of veterinarians and farmers to those farming practices suspected to favor bTB transmission.

Further studies will be needed to ascertain possible relationships between these farming practices and the spread of bTB in livestock, and to identify risk factors for infection, which would ultimately inform the implementation of targeted surveillance and control measures, especially in the ecosystem-wildlife-livestock-human interface areas.

## Supporting information

S1 Table Combined genetic profiles (N = 73) of 204 *M*. *bovis* isolates. (DOCX)

S1 Fig Minimum spanning tree showing SB0120/MIRU-VNTR combined genetic profiles. (TIF)

S2 Fig Minimum spanning tree showing SB0841/MIRU-VNTR combined genetic profiles. (TIF)

S3 Fig Minimum spanning tree showing SB0134/MIRU-VNTR combined genetic profiles. (TIF)

## References

[1] Biet F, Boschiroli ML, Thorel MF, Guilloteau LA. Zoonotic aspects of *Mycobacterium bovis* and Mycobacterium avium-intracellulare complex (MAC). Vet Res. 2005; 36: 411–436. 10.1051/vetres:2005001 15845232

[2] El-Sayed A, El-Shannat S, Kamel M, Castañeda-Vazquez MA, Castañeda-Vazquez H. Molecular epidemiology of *Mycobacterium bovis* in humans and cattle. Zoonoses Public Health 2016; 63: 251–264. 10.1111/zph.12242 26684712

[3] Palmer MV. *Mycobacterium bovis*: characteristics of wildlife reservoir hosts. Transbound Emerg Dis. 2013; 60 Suppl 1: 1–13. 10.1111/tbed.12115 24171844

[4] Buddle BM, de Lisle GW, Griffin JF, Hutchings SA. Epidemiology, diagnostics, and management of tuberculosis in domestic cattle and deer in New Zealand in the face of a wildlife reservoir. N Z Vet J. 2015; 63 Suppl 1: 19–27. 24992203 10.1080/00480169.2014.929518 PMC4566881

[5] O’Reilly LM, Daborn CJ. The epidemiology of *Mycobacterium bovis* infections in animals and man: a review. Tuber Lung Dis. 1995; 76 Suppl 1:1–46. 10.1016/0962-8479(95)90591-x 7579326

[6] Grange JM. *Mycobacterium bovis* infection in human beings. Tuberculosis 2001; 81: 71–77. 10.1054/tube.2000.0263 11463226

[7] Pérez-Lago L, Navarro Y, García-de-Viedma D. Current knowledge and pending challenges in zoonosis caused by *Mycobacterium bovis*: a review. Res Vet Sci. 2014; 97 Suppl: S94–S100. 24360647 10.1016/j.rvsc.2013.11.008

[8] Michel AL, Muller B, Van Helden PD. *Mycobacterium bovis* at the animal-human interface: a problem, or not? Vet Microbiol. 2010; 140: 371–381. 10.1016/j.vetmic.2009.08.029 19773134

[9] Olea-Popelka F, Muwonge A, Perera A, Dean AS, Mumford E, Erlacher-Vindel E, et al Zoonotic tuberculosis in human beings caused by *Mycobacterium bovis*—a call for action. Lancet Infect Dis. 2017; 17: e21–e25. 10.1016/S1473-3099(16)30139-6 27697390

[10] Kamerbeek J, Schouls L, Kolk A, van Agterveld M, van Soolingen D, Kuijper S, et al Simultaneous detection and strain differentiation of *Mycobacterium tuberculosis* for diagnosis and epidemiology. J Clin Microbiol. 1997; 35: 907–914. 9157152 10.1128/jcm.35.4.907-914.1997 PMC229700

[11] Supply P, Magdalena J, Himpens S, Locht C. Identification of novel intergenic repetitive units in a mycobacterial two-component system operon. Mol Microbiol. 1997; 26: 991–1003. 9426136 10.1046/j.1365-2958.1997.6361999.x

[12] Yun KW, Song EJ, Choi GE, Hwang IK, Lee EY, Chang CL. Strain typing *of Mycobacterium tuberculosis* isolates from Korea by mycobacterial interspersed repetitive units-variable number of tandem repeats. Korean J Lab Med. 2009; 29: 314–319. 10.3343/kjlm.2009.29.4.314 19726893

[13] Boniotti MB, Goria M, Loda D, Garrone A, Benedetto A, Mondo A, et al Molecular typing of *Mycobacterium bovis* strains isolated in Italy from 2000 to 2006 and evaluation of variable-number tandem repeats for geographically optimized genotyping. J Clin Microbiol. 2009; 47: 636–644. 10.1128/JCM.01192-08 19144792 PMC2650904

[14] Rodriguez-Campos S, Navarro Y, Romero B, de Juan L, Bezos J, Mateos A, et al Splitting of a prevalent *Mycobacterium bovis* spoligotype by variable-number tandem-repeat typing reveals high heterogeneity in an evolving clonal group. J Clin Microbiol. 2013; 51: 3658–3665. 10.1128/JCM.01271-13 23985914 PMC3889748

[15] Armas F, Camperio C, Coltella L, Selvaggini S, Boniotti MB, Pacciarini ML, et al Comparison of semi-automated commercial rep-PCR fingerprinting, spoligotyping, 12-locus MIRU-VNTR typing and single nucleotide polymorphism analysis of the *embB* gene as molecular typing tools for *Mycobacterium bovis*. J Med Microbiol. 2017 8 4 10.1099/jmm.0.000536 28771142

[16] Hauer A, De Cruz K, Cochard T, Godreuil S, Karoui C, Henault S, et al Genetic evolution of *Mycobacterium bovis* causing tuberculosis in livestock and wildlife in France since 1978. PLoS One. 2015 2 6 10.1371/journal.pone.0117103 25658691 PMC4319773

[17] Carvalho RC, Vasconcellos SE, Issa Mde A, Soares Filho PM, Mota PM, Araújo FR, et al Molecular typing of *Mycobacterium bovis* from cattle reared in Midwest Brazil. PLoS One. 2016 9 15 10.1371/journal.pone.0162459 27631383 PMC5024986

[18] Egbe NF, Muwonge A, Ndip L, Kelly RF, Sander M, Tanya V, et al Molecular epidemiology of *Mycobacterium bovis* in Cameroon. Sci Rep. 2017 7 5 10.1038/s41598-017-04230-6 28680043 PMC5498612

[19] Machado A, Rito T, Ghebremichael S, Muhate N, Maxhuza G, Macuamule C, et al Genetic diversity and potential routes of transmission of *Mycobacterium bovis* in Mozambique. PLoS Negl Trop Dis. 2018 1 18 10.1371/journal.pntd.0006147 29346413 PMC5772998

[20] Di Marco V, Mazzone P, Capucchio MT, Boniotti MB, Aronica V, Russo M, et al Epidemiological significance of the domestic black pig (Sus scrofa) in maintenance of bovine tuberculosis in Sicily. J Clin Microbiol. 2012; 50: 1209–1218. 10.1128/JCM.06544-11 22322347 PMC3318573

[21] Amato B, Di Marco Lo Presti V, Gerace E, Capucchio MT, Vitale M, Zanghì P, et al Molecular epidemiology of *Mycobacterium tuberculosis* complex strains isolated from livestock and wild animals in Italy suggests the need for a different eradication strategy for bovine tuberculosis. Transbound Emerg Dis. 2018 4 10.1111/tbed.12776 29205877

[22] Frothingham R, Meeker-O'Connell WA. Genetic diversity in the *Mycobacterium tuberculosis* complex based on variable numbers of tandem DNA repeats. Microbiology 1998; 144: 1189–1196. 10.1099/00221287-144-5-1189 9611793

[23] Supply P, Mazars E, Lesjean S, Vincent V, Gicquel B, Locht C. Variable human minisatellite-like regions in the *Mycobacterium tuberculosis* genome. Mol Microbiol. 2000; 36: 762–771. 10844663 10.1046/j.1365-2958.2000.01905.x

[24] Skuce RA, McCorry TP, McCarroll JF, Roring SM, Scott AN, Brittain D, et al Discrimination of *Mycobacterium tuberculosis* complex bacteria using novel VNTR-PCR targets. Microbiology 2002; 148: 519–528. 10.1099/00221287-148-2-519 11832515

[25] Roring S, Scott A, Brittain D, Walker I, Hewinson G, Neill S, et al Development of variable-number tandem repeat typing of *Mycobacterium bovis*: comparison of results with those obtained by using existing exact tandem repeats and spoligotyping. J Clin Microbiol. 2002; 40: 2126–2133. 10.1128/JCM.40.6.2126-2133.2002 12037076 PMC130792

[26] Ghavidel M, Mansury D, Nourian K, Ghazvini K. The most common spoligotype of *Mycobacterium bovis* isolated in the world and the recommended loci for VNTR typing; A systematic review. Microb Pathog. 2018; 118: 310–315. 10.1016/j.micpath.2018.03.036 29578066

[27] Probst C, Freuling C, Moser I, Geue L, Köhler H, Conraths FJ, et al Bovine tuberculosis: making a case for effective surveillance. Epidemiol Infect. 2011; 139: 105–112. 10.1017/S0950268810000786 20392304

[28] Broughan JM, Judge J, Ely E, Delahay RJ, Wilson G, Clifton-Hadley RS, et al A review of risk factors for bovine tuberculosis infection in cattle in the UK and Ireland. Epidemiol Infect. 2016; 144: 2899–2926. 10.1017/S095026881600131X 27452974 PMC9150416

[29] Marsot M, Béral M, Scoizec A, Mathevon Y, Durand B, Courcoul A. Herd-level risk factors for bovine tuberculosis in French cattle herds. Prev Vet Med. 2016; 131: 31–40. 10.1016/j.prevetmed.2016.07.006 27544249

