# Supplementary material for: Genotype diversity and distribution of Mycobacterium bovis from livestock in a small, high-risk area in northeastern Sicily, Italy
Source: PLoS Negl Trop Dis. 2019 Jul 15;13(7):e0007546. doi: 10.1371/journal.pntd.0007546 (PMC6658142; doi:10.1371/journal.pntd.0007546)
Supplement: S1 Table — (DOCX) [file pntd.0007546.s001.docx]

**S1 Table. Combined genetic profiles (N=73) of 204 *M. bovis* isolates**

| Spoligotype/MIRU-VNTR type | Profile* | (n_c_, n_p_) |
| --- | --- | --- |
| SB0120/4 5 5 3 3 10 4 4 4 3 6 5  SB0120/3 3 5 3 3 10 6 4 4 3 6 5  SB0120/3 3 5 3 3 10 4 4 4 3 6 5  SB0120/5 5 5 3 4 10 4 5 4 3 6 5  SB0120/4 5 5 3 3 10 4 4 2 3 6 5  SB0120/4 5 5 3 3 10 4 3 4 3 6 5  SB0120/4 5 5 3 3 7 4 4 4 3 6 5  SB0120/4 5 5 3 3 10 4 2 4 3 6 5  SB0120/5 3 5 3 4 10 4 5 4 3 6 5  SB0120/3 3 5 3 3 10 4 4 4 3 6 4  SB0120/5 3 5 3 3 10 4 4 3 3 6 5  SB0120/5 4 5 3 3 10 4 4 4 1 7 5  SB0120/4 3 5 3 3 10 4 4 4 3 6 5  SB0120/4 4 5 3 3 10 4 4 4 3 6 5  SB0120/4 5 5 3 3 10 3 4 4 3 6 5  SB0120/4 5 5 3 3 10 3 4 2 3 6 5  SB0120/4 5 5 3 3 10 5 4 4 3 6 5  SB0120/4 5 5 3 3 10 4 4 4 3 6 4  SB0120/5 3 5 3 3 10 4 4 4 3 6 5  SB0120/3 3 5 3 3 10 3 4 4 3 6 5  SB0120/3 3 5 3 3 10 4 4 4 3 7 5  SB0120/3 3 5 3 3 10 6 4 4 3 7 5  SB0120/3 3 5 3 3 10 6 4 4 2 6 5  SB0120/3 3 5 3 3 10 6 2 4 3 6 5  SB0120/5 3 5 3 4 10 4 1 4 3 6 5  SB0120/5 4 5 3 3 10 4 4 4 3 6 5  SB0120/5 4 5 3 3 10 4 3 4 3 6 5  SB0120/5 5 5 3 3 10 4 4 4 3 5 5  SB0120/5 5 6 3 3 10 4 4 4 3 5 5  SB0120/5 5 6 3 3 10 4 4 4 3 8 5  SB0120/5 5 5 3 4 10 4 1 4 3 6 5  SB0120/5 5 5 3 4 10 4 5 4 2 6 5  SB0120/6 4 5 3 3 10 2 5 4 3 6 2  SB0841/5 5 5 3 3 10 4 4 4 3 5 5  SB0841/5 5 5 3 3 10 4 4 4 3 6 5  SB0841/5 5 5 3 3 5 4 4 4 3 6 5  SB0841/5 4 5 3 3 10 4 4 4 3 6 5  SB0841/5 5 5 3 3 10 4 4 4 3 5 6  SB0841/5 5 5 3 3 10 4 4 4 3 5 4  SB0841/4 4 5 3 3 10 4 4 4 3 6 5  SB0841/4 5 5 3 3 10 4 4 2 3 6 5  SB0841/5 4 5 3 3 10 4 1 4 3 6 5  SB0841/5 4 5 3 3 7 4 4 4 3 6 5  SB0841/5 4 5 3 3 10 4 4 4 3 7 5  SB0841/5 5 5 3 3 10 4 4 4 3 6 6  SB0841/5 5 5 3 3 10 4 1 4 3 6 5  SB0841/5 5 5 3 3 10 4 4 4 1 5 5  SB0841/5 5 2 3 3 10 4 4 4 3 6 4  SB0841/6 4 5 3 3 10 4 4 4 3 6 6  SB0134/5 4 5 3 4 10 3 5 4 3 6 5  SB0134/5 4 5 3 4 10 4 5 4 3 6 5  SB0134/5 4 5 3 4 10 2 5 4 3 6 5  SB0134/5 4 5 2 4 10 4 5 4 3 6 5  SB0134/5 4 5 3 4 10 4 5 4 3 8 5  SB0134/5 4 5 3 4 10 4 5 4 3 6 7  SB0134/5 4 5 3 3 10 3 5 4 3 6 5  SB0134/5 4 5 2 4 10 4 5 4 3 5 5  SB0134/5 4 5 2 4 10 4 5 4 3 5 6  SB0134/5 4 5 3 4 10 3 6 4 2 6 5  SB0850/4 5 5 3 3 10 4 4 3 3 5 5  SB2473/6 4 3 3 3 10 2 5 4 3 7 2  SB0121/6 4 3 3 3 10 2 9 4 3 9 2  SB0121/6 4 3 3 3 10 2 5 4 3 9 2  SB0822/5 4 5 3 3 10 4 4 4 1 6 5  SB0133/5 5 5 3 3 10 4 4 4 3 6 5  SB1564/3 3 5 3 3 10 4 4 4 3 6 5  SB1564/2 3 5 3 3 10 4 4 4 3 6 5  SB0961/5 5 5 3 3 10 3 4 4 3 5 5  SB1305/6 4 3 3 3 10 2 5 4 3 7 2  SB1565/5 5 5 3 3 10 4 4 4 3 6 5  SB1566/3 3 5 3 3 10 4 4 4 3 6 5  SB1570/4 5 5 3 3 10 4 3 4 3 6 5  SB1572/4 5 5 3 3 10 4 2 4 3 6 5 | SB0120/a  SB0120/b  SB0120/c  SB0120/d  SB0120/e  SB0120/f  SB0120/g  SB0120/h  SB0120/i  SB0120/j  SB0120/k  SB0120/l  -  -  -  -  -  -  -  -  -  -  -  -  -  -  -  -  -  -  -  -  -  SB0841/m  SB0841/n  SB0841/o  SB0841/p  SB0841/q  SB0841/r  -  -  -  -  -  -  -  -  -  -  SB0134/s  SB0134/t  SB0134/u  SB0134/v  -  -  -  -  -  -  -  -  -  -  -  -  -  -  -  -  -  -  -  - | (22, 5)  (15, 1)  (5, 2)  (7, 0)  (1, 4)  (3, 1)  (3, 0)  (3, 0)  (3, 0)  (1, 1)  (0, 2)  (2, 0)  (1, 0)  (1, 0)  (1, 0)  (1, 0)  (1, 0)  (1, 0)  (1, 0)  (1, 0)  (1, 0)  (1, 0)  (1, 0)  (1, 0)  (1, 0)  (1, 0)  (1, 0)  (0, 1)  (1, 0)  (1, 0)  (1, 0)  (1, 0)  (1, 0)  (4, 11)  (3, 8)  (1, 1)  (2, 0)  (2, 0)  (0, 2)  (1, 0)  (1, 0)  (1, 0)  (1, 0)  (1, 0)  (1, 0)  (1, 0)  (0, 1)  (1, 0)  (1, 0)  (11, 0)  (9, 0)  (4, 0)  (2, 0)  (1, 0)  (1, 0)  (1, 0)  (1, 0)  (1, 0)  (1, 0)  (5, 0)  (5, 0)  (1, 0)  (2, 0)  (3, 0)  (2, 0)  (1, 0)  (1, 0)  (1, 0)  (1, 0)  (1, 0)  (1, 0)  (1, 0)  (1, 0) |

*Genotypes obtained by combining the predominant spoligotypes SB0120, SB0841 and SB013 and MIRU-VNTR results and common to at least two animals are marked.

n_c_ = number of isolates from cattle; n_p_ = number of isolates from black pigs.
