# Supplementary figures and images for: Genotype diversity and distribution of Mycobacterium bovis from livestock in a small, high-risk area in northeastern Sicily, Italy
Source: PLoS Negl Trop Dis. 2019 Jul 15;13(7):e0007546. doi: 10.1371/journal.pntd.0007546 (PMC6658142; doi:10.1371/journal.pntd.0007546)

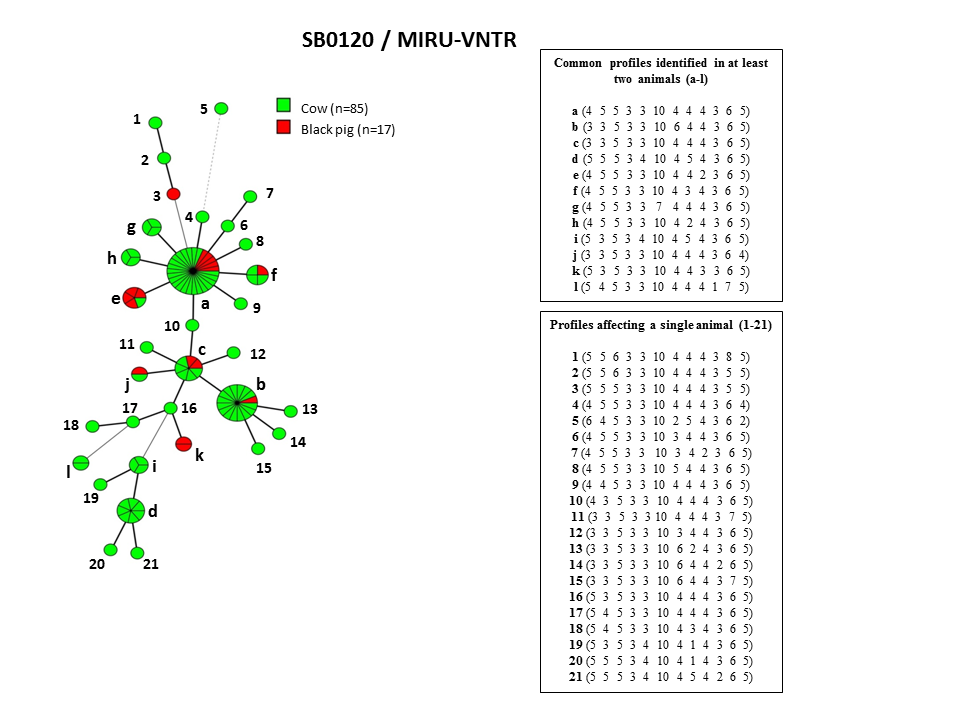

Supplement: S1 Fig — (TIF) [file pntd.0007546.s002.tif]

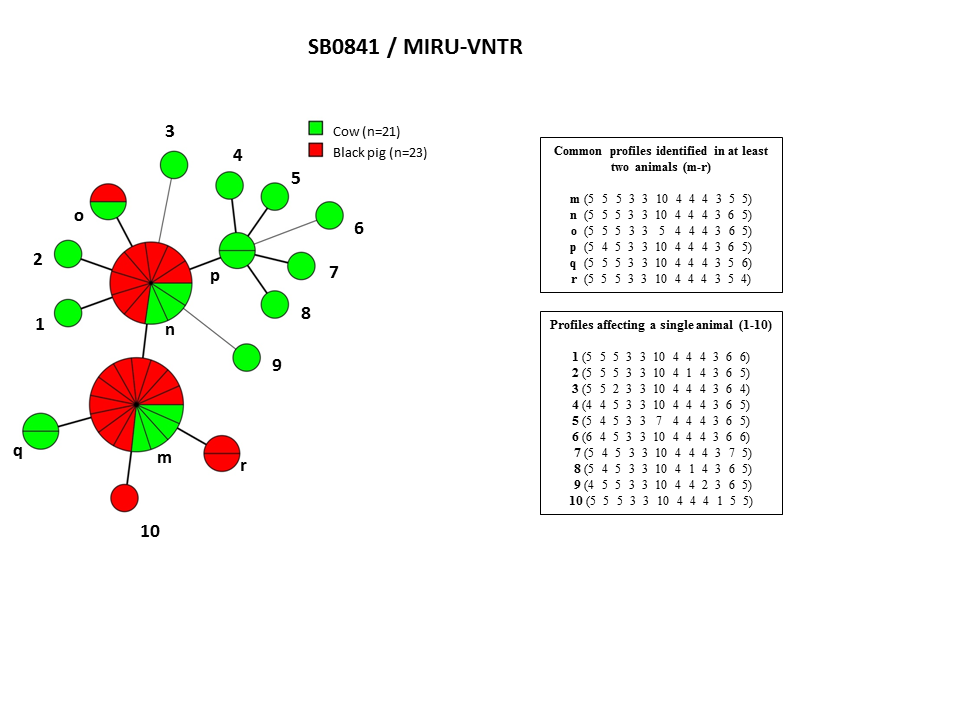

Supplement: S2 Fig — (TIF) [file pntd.0007546.s003.tif]

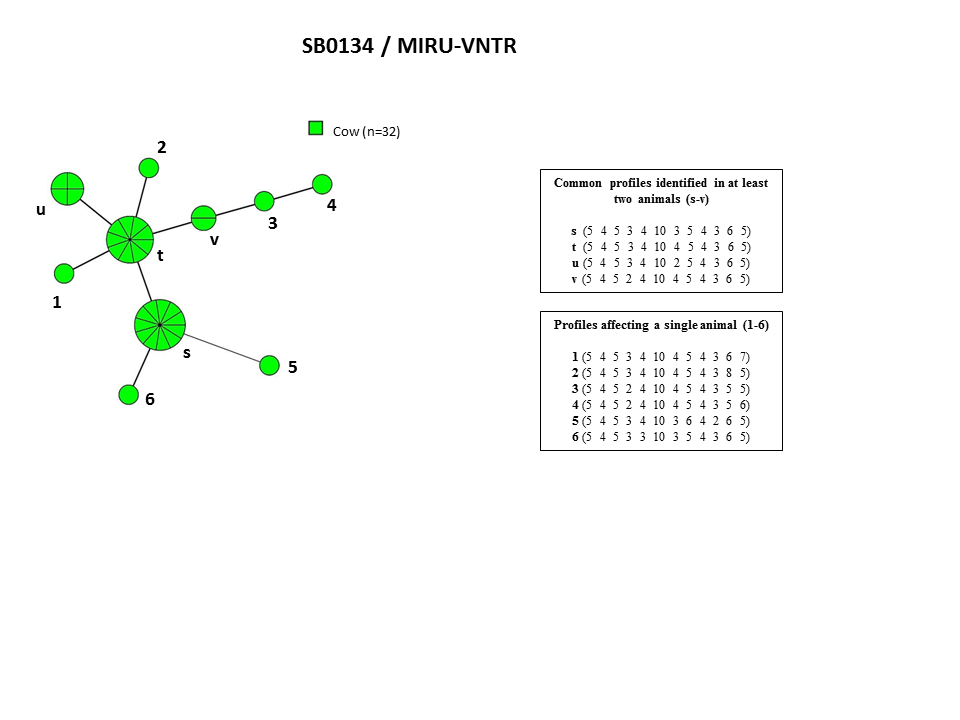

Supplement: S3 Fig — (TIF) [file pntd.0007546.s004.tif]
